# Effects of Dapagliflozin in Patients in Asia: A Post Hoc Subgroup Analysis From the DELIVER Trial

**DOI:** 10.1016/j.jacasi.2023.10.005

**Published:** 2023-12-05

**Authors:** Xiaowen Wang, Carolyn S.P. Lam, Muthiah Vaduganathan, Toru Kondo, Mingming Yang, Yaling Han, Vinh Nguyen Pham, Chern-En Chiang, Masafumi Kitakaze, Zi Michael Miao, Pardeep S. Jhund, Akshay S. Desai, Silvio E. Inzucchi, Rudolf A. de Boer, Felipe A. Martinez, Mikhail N. Kosiborod, Adrian F. Hernandez, Brian Claggett, Anna Maria Langkilde, John J.V. McMurray, Scott D. Solomon

**Affiliations:** a Division of Cardiovascular Medicine, Brigham and Women’s Hospital and Harvard Medical School, Boston, Massachusetts, USA; b National Heart Centre Singapore, Duke-NUS Medical School, Singapore, Singapore; c British Heart Foundation Cardiovascular Research Centre, University of Glasgow, Glasgow, Scotland, United Kingdom; d Department of Cardiology, Nagoya University Graduate School of Medicine, Nagoya, Japan; e Department of Cardiology, Zhongda Hospital, School of Medicine, Southeast University, Nanjing, China; f Cardiovascular Research Institute and Department of Cardiology, General Hospital of Northern Theater Command, Shenyang, China; g Department of Internal Medicine, Tan Tao University, Tan Duc, Vietnam; h General Clinical Research Center and Division of Cardiology, Taipei Veterans General Hospital and National Yang-Ming University, Taipei, Taiwan; i Hanwa Memorial Hospital, Osaka, Japan; j The Osaka Medical Research Foundation for Intractable Diseases, Osaka, Japan; k Section of Endocrinology, Yale University School of Medicine, New Haven, Connecticut, USA; l Erasmus Medical Center, Department of Cardiology, Rotterdam, the Netherlands; m National University of Cordoba, Cordoba, Argentina; n Department of Cardiology, Saint Luke’s Mid America Heart Institute, University of Missouri-Kansas City, Kansas City, Missouri, USA; o Duke Clinical Research Institute, Duke University Medical Center, Durham, North Carolina, USA; p Late-Stage Development, Cardiovascular, Renal, and Metabolism, BioPharmaceuticals R&D, AstraZeneca, Gothenburg, Sweden

**Keywords:** clinical trials, heart failure, HFmrEF, HFpEF, SGLT2 inhibitor

## Abstract

**Background:**

Patients with heart failure (HF) with mildly reduced or preserved ejection fraction in Asia may have different clinical characteristics and outcomes compared with patients from other parts of the world.

**Objectives:**

The purpose of this study was to investigate the clinical characteristics, safety, and efficacy of dapagliflozin in patients in Asia vs outside Asia in the DELIVER (Dapagliflozin Evaluation to Improve the Lives of Patients with Preserved Ejection Fraction Heart Failure) trial.

**Methods:**

In the DELIVER trial, patients with HF and left ventricular ejection fraction >40% were enrolled across 353 sites in 20 countries. The effects of dapagliflozin vs placebo on primary (composite of worsening HF or cardiovascular death) and secondary outcomes were compared in patients from Asia vs outside Asia.

**Results:**

Among 6,263 participants, 1,226 (19.6%) were enrolled in Asia. Participants from Asia were less likely to have diabetes, hypertension, history of myocardial infarction, or obesity. After adjusting for clinically relevant characteristics, those in Asia had similar risks of primary composite outcome compared with those from outside Asia (HR: 0.97; 95% CI: 0.82-1.15). Those in Asia had a lower risk of all-cause mortality compared with those enrolled outside Asia (HR: 0.54; 95% CI: 0.44-0.66). Enrollment from Asia did not modify the effect of dapagliflozin on the primary outcome (*P*_interaction_ = 0.54). Serious adverse events and rates of drug discontinuation were also balanced in both treatment arms, irrespective of enrollment in Asia vs outside Asia.

**Conclusions:**

In the global DELIVER trial, dapagliflozin reduced the risk of CV death or worsening HF events and was well tolerated among participants enrolled in both Asia and other geographic regions.

Asia comprises 60% of the world's population and is both ethnically and socioeconomically diverse. With a rapidly aging population, urbanization, and increased prevalence of comorbidities such as diabetes, obesity, and hypertension, heart failure (HF) has become an urgent public health concern in this region.^1^

Epidemiological data suggest that many Asian countries have worse HF outcomes compared with Western countries, but with significant variation among nations. For example, in the INTER-CHF (International Congestive Heart Failure) study, India had the highest 1-year mortality (23%), compared with Southeast Asia (15%) and China (7%).^2^ Data from the ASIAN-HF (Asian Sudden Cardiac Death in Heart Failure) registry showed the highest mortality in Southeast Asia.^3^ Moreover, comorbidities and outcomes vary enormously between and even within Asian countries and ethnicities.

Yet despite its large population with high rates of adverse outcomes, Asian countries have been generally underrepresented in global clinical trials until recently. For example, most of the pivotal trials for angiotensin-converting enzyme (ACE) inhibitors and beta blockers did not include any Asian countries at all.^4^ The DELIVER (Dapagliflozin Evaluation to Improve the Lives of Patients with Preserved Ejection Fraction Heart Failure) trial included a fifth of participants from Asia,^5^^,^^6^ thus providing a unique opportunity to study the characteristics, outcomes, and response to therapy in a contemporary cohort of patients with HF from Asia.

## Methods

### Study design and patient population

The design of DELIVER has been previously reported.^5^^,^^7^ Briefly, 6,263 patients were enrolled in the phase III, international, double-blind, randomized-controlled trial. Patients with chronic HF and left ventricular ejection fraction (LVEF) >40% (including previous LVEF ≤40%) were randomized to receive dapagliflozin 10 mg daily or a matching placebo. In addition to LVEF, other key inclusion criteria included NYHA functional class II-IV HF, evidence of structural heart disease on echocardiography (left atrial enlargement or left ventricular hypertrophy), and elevated natriuretic peptides (NT-proBNP [N-terminal pro–B-type natriuretic peptides] ≥300 pg/mL for those without atrial fibrillation/flutter, or ≥600 pg/mL for those in atrial fibrillation/flutter). Patients were followed for a median of 2.3 years. The trial protocol for DELIVER were approved by institutional review boards at each trial center and trial participants gave informed consent.

### Definition of regions and ethnicities

In the DELIVER trial, the prespecified geographic regions included Asia, Latin America, North America, and Europe and Saudi Arabia. Participants enrolled from Asia were from Japan, China, Taiwan, and Vietnam.

### Outcomes

The primary outcome for the DELIVER trial was a composite of worsening HF or cardiovascular (CV) death, analyzed according to intention-to-treat principles consistent with the DELIVER statistical analysis plan. Worsening HF was defined as HF hospitalization or urgent visit for HF.^5^^,^^7^ Secondary outcomes included total number of worsening HF and CV death, change from baseline in Kansas City Cardiomyopathy Questionnaire (KCCQ) total symptom score at 8 months, CV death, and all-cause death.

Key safety outcomes included serious adverse events, adverse events, adverse events leading to drug discontinuations or dose interruption, and select adverse outcomes such as hypoglycemia and diabetic ketoacidosis.

### Statistical analysis

Baseline characteristics for those enrolled in Asia vs outside Asia were compared. Continuous variables were analyzed using Student’s *t*-test and reported as mean ± SD. Non-normally distributed variables were analyzed using Wilcoxon rank-sum test. For comparisons between Asian countries, analysis of variance and Kruskal-Wallis tests were used for normally and non-normally distributed continuous variables, respectively. Categorical variables were compared using chi-square test and reported as numbers and percentages. Cox regression models were used to compare outcomes in those enrolled in Asia vs outside Asia. Three models were constructed: 1) unadjusted; 2) with age, sex, and baseline LVEF as covariates; and 3) with body mass index (BMI), NYHA functional class, atrial fibrillation/flutter, stroke, dyslipidemia, diabetes, myocardial infarction, hypertension, and prior HF hospitalization as covariates in addition to those included in Model 2. Total events analyses were performed using the Lin-Wei-Yang-Ying model.

To evaluate the effect of dapagliflozin in participants in Asia vs outside Asia, we constructed Cox proportional hazards models without covariates. To assess the impact of region on the treatment effect of dapagliflozin, we included a region-by-treatment interaction term in the Cox proportional hazards models.

Linear regression was used to compare changes in KCCQ scores at 8 months, with baseline KCCQ included in the linear regression model to account for baseline differences. The impact of region was tested by including a region-by-treatment interaction term in the linear regression model. Stata, version 16 (StataCorp) was used for all analyses. The value *P* < 0.05 was considered statistically significant. No adjustments were made for multiple comparisons.

## Results

### Baseline characteristics

There were 1,226 (19.6%) participants enrolled from Asia, all of whom were of Asian ethnicity. Among the participants who were enrolled from outside Asia, most were White (88.1%), with 48 participants of Asian ethnicity (1%). At baseline, those enrolled in Asia were of similar age as those enrolled outside of Asia but were more likely to be men (Table 1). Those enrolled in Asia had a lower burden of comorbidities, as evidenced by less obesity, dyslipidemia, type 2 diabetes, hypertension, myocardial infarction, stroke, chronic obstructive pulmonary disease, and sleep apnea (Table 1). Compared with those enrolled from outside Asia, those enrolled in Asia had similar levels of NT-proBNP, higher baseline LVEF, were less likely to have NYHA functional class III/IV symptoms, but were more likely to have a history of HF hospitalization. Participants from Asia were less likely to be on loop diuretics, ACE inhibitors, and beta blockers, but were more likely to be on angiotensin receptor blockers and mineralocorticoid receptor antagonists.

**Table 1:** Baseline Characteristics of Patients Enrolled in Asia vs Outside Asia

	Outside Asia (n = 5,037)	Asia (n = 1,226)	Asia vs Outside Asia *P* Value	China (n = 310)	Taiwan (n = 318)	Japan (n = 422)	Vietnam (n = 176)	Within Asia *P* Value
Age, y	71.7 ± 9.3	71.4 ± 10.4	0.25	66.0 ± 9.6	72.8 ± 10.6	75.3 ± 9.0	69.0 ± 9.9	<0.001
Male	2,765 (54.9)	751 (61.3)	<0.001	206 (66.5)	180 (56.6)	280 (66.4)	85 (48.3)	<0.001
Race			<0.001					—
White	4,439 (88.1)	0 (0.0)		0 (0.0)	0 (0.0)	0 (0.0)	0 (0.0)	
Asian	48 (1.0)	1,226 (100.0)		310 (100.0)	318 (100.0)	422 (100.0)	176 (100.0)	
Black or African American	159 (3.2)	0 (0.0)		0 (0.0)	0 (0.0)	0 (0.0)	0 (0.0)	
American Indian or Alaska Native	189 (3.8)	0 (0.0)		0 (0.0)	0 (0.0)	0 (0.0)	0 (0.0)	
Other	202 (4.0)	0 (0.0)		0 (0.0)	0 (0.0)	0 (0.0)	0 (0.0)	
Baseline medical history								
AFF	2,829 (56.2)	723 (59.0)	0.08	147 (47.4)	221 (69.5)	266 (63.0)	89 (50.6)	<0.001
Stroke	458 (9.1)	139 (11.3)	0.016	30 (9.7)	42 (13.2)	62 (14.7)	5 (2.8)	<0.001
Dyslipidemia	3,320 (65.9)	670 (54.6)	<0.001	67 (21.6)	185 (58.2)	276 (65.4)	142 (80.7)	<0.001
Type 2 diabetes mellitus	2,329 (46.2)	477 (38.9)	<0.001	131 (42.3)	144 (45.3)	152 (36.0)	50 (28.4)	<0.001
Chronic obstructive pulmonary disease	618 (12.3)	74 (6.0)	<0.001	8 (2.6)	47 (14.8)	18 (4.3)	1 (0.6)	<0.001
Sleep apnea	440 (8.7)	45 (3.7)	<0.001	5 (1.6)	12 (3.8)	27 (6.4)	1 (0.6)	<0.001
Myocardial infarction	1,375 (27.3)	264 (21.5)	<0.001	80 (25.8)	46 (14.5)	98 (23.2)	40 (22.7)	0.003
Hypertension	4,610 (91.5)	943 (76.9)	<0.001	207 (66.8)	262 (82.4)	343 (81.3)	131 (74.4)	<0.001
Prior HF hospitalization	1,983 (39.4)	556 (45.4)	<0.001	192 (61.9)	94 (29.6)	218 (51.7)	52 (29.5)	<0.001
Any coronary artery disease	2,584 (51.3)	634 (51.7)	0.80	165 (53.2)	173 (54.4)	200 (47.4)	96 (54.5)	0.18
Any atherosclerotic cardiovascular disease	2,886 (57.3)	712 (58.1)	0.62	186 (60.0)	198 (62.3)	229 (54.3)	99 (56.2)	0.14
Never smoker	2,845 (56.5)	673 (54.9)	0.24	187 (60.3)	196 (61.6)	173 (41.0)	117 (66.5)	<0.001
Overweight or obese	4,293 (85.3)	567 (46.3)	<0.001	157 (50.6)	192 (60.6)	162 (38.4)	56 (31.8)	<0.001
Body mass index	31.0 ± 6.0	25.1 ± 4.2	<0.001	25.4 ± 3.7	26.4 ± 4.6	24.4 ± 4.0	23.8 ± 4.1	<0.001
NYHA functional class III/IV	1353 (26.8)	196 (16.0)	<0.001	213 (36.5)	48 (15.1)	25 (5.9)	10 (5.7)	<0.001
LVEF (%)	53.8 ± 8.4	55.7 ± 9.8	<0.001	50.6 ± 7.7	57.5 ± 9.8	57.5 ± 9.4	57.4 ± 10.9	<0.001
NT-proBNP (ng/L)	1,010 (616–1,754)	1,016 (656–1,746)	0.62	1,061 (614–1,851)	979 (668–1,677)	1,044 (649–1,735)	990 (664–1,504)	0.54
NT-proBNP in AFF	1,408 (970–2,244)	1,378 (925–2,149)	0.17	1,576 (1,054–2,261)	1,279 (902–2,060)	1,487 (933–2,271)	1,214 (933–1,793)	0.14
NT-proBNP when no AFF	709 (468–1,292)	731 (479–1,252)	0.92	762 (484–1395)	704 (444–1150)	737 (501–1,269)	737 (440–1,139)	0.39
Systolic blood pressure (mm Hg)	129.1 ± 14.8	124.5 ± 16.9	<0.001	120.7 ± 16.2	125.4 ± 16.5	128.2 ± 16.9	120.9 ± 16.9	<0.001
Diastolic blood pressure (mm Hg)	74.4 ± 10.1	72.2 ± 11.2	<0.001	72.9 ± 10.6	73.1 ± 11.8	71.9 ± 11.6	69.8 ± 10.0	0.010
HbA1c (%)	6.6 ± 1.4	6.4 ± 1.2	<0.001	6.6 ± 1.5	6.5 ± 1.2	6.2 ± 0.8	6.4 ± 1.3	<0.001
Pulse (beats/min)	71.2 ± 11.5	72.7 ± 12.5	<0.001	71.9 ± 12.4	74.1 ± 12.5	71.3 ± 11.8	75.3 ± 14.0	<0.001
Creatinine (μmol/L)	102.3 ± 31.3	103.1 ± 30.3	0.40	99.2 ± 29.1	109.6 ± 34.6	100.7 ± 27.7	104.4 ± 28.1	<0.001
eGFR (mL/min/1.73 m^2^)	61.0 ± 19.2	61.2 ± 19.1	0.77	67.1 ± 20.1	56.5 ± 18.1	61.4 ± 18.4	58.7 ± 17.8	<0.001
eGFR <60 mL/min/1.73 m^2^	2,464 (48.9)	606 (49.4)	0.75	118 (38.1)	181 (56.9)	212 (50.2)	95 (54.0)	<0.001
Baseline medications								
Loop diuretics	3,969 (78.8)	842 (68.7)	<0.001	236 (76.1)	162 (50.9)	336 (79.6)	108 (61.4)	<0.001
ACE inhibitor	2,086 (41.4)	209 (17.0)	<0.001	44 (14.2)	16 (5.0)	125 (29.6)	24 (13.6)	<0.001
Angiotensin receptor blocker	1,742 (34.6)	530 (43.2)	<0.001	91 (29.4)	161 (50.6)	165 (39.1)	113 (64.2)	<0.001
Angiotensin receptor neprilysin inhibitor	178 (3.5)	123 (10.0)	<0.001	89 (28.7)	33 (10.4)	0 (0.0)	1 (0.6)	<0.001
Beta blocker	4,243 (84.2)	934 (76.2)	<0.001	249 (80.3)	227 (71.4)	331 (78.4)	127 (72.2)	0.021
Mineralocorticoid receptor antagonist	2,038 (40.5)	629 (51.3)	<0.001	219 (70.6)	140 (44.0)	155 (36.7)	115 (65.3)	<0.001

Values are mean ± SD, n (%), or median (IQR), unless otherwise noted.

ACE = angiotensin-converting enzyme; AFF = atrial fibrillation/flutter; eGFR = estimated glomerular filtration rate; HbA1c = hemoglobin A1c; HF = heart failure; LVEF = left ventricular ejection fraction; NT-proBNP = N-terminal pro–B-type brain natriuretic peptide.

Within Asia, patients from China were the youngest (mean age 66 ± 10 years), and patients from Japan were the oldest (mean age 75 ± 9 years) (Table 1). The proportion of patients who were men was highest in China (66.5%) and lowest in Vietnam (48.3%). Dyslipidemia was highly prevalent in Vietnam (80.7%) compared with Japan (65.4%), Taiwan (58.2%), and China (21.6%). Diabetes was more prevalent in Taiwan (45.3%) and China (42.3%) than Japan (36.0%) and Vietnam (28.4%). Baseline NT-proBNP was similar in countries of Asia. Patients from China most often reported a prior hospitalization for HF (61.9%) compared with least often in Vietnam (29.5%). Those enrolled from China had the highest prevalence of NYHA functional class III/IV symptoms (36.5%) compared with those enrolled from Vietnam with the lowest prevalence (5.7%). Patients from China had the lowest baseline LVEF (51% ± 8%) compared with Taiwan (58% ± 10%), Japan (58% ± 10%), and Vietnam (57% ± 11%) (Table 1).

### Outcomes in Asia vs outside Asia

**Figure 1:**
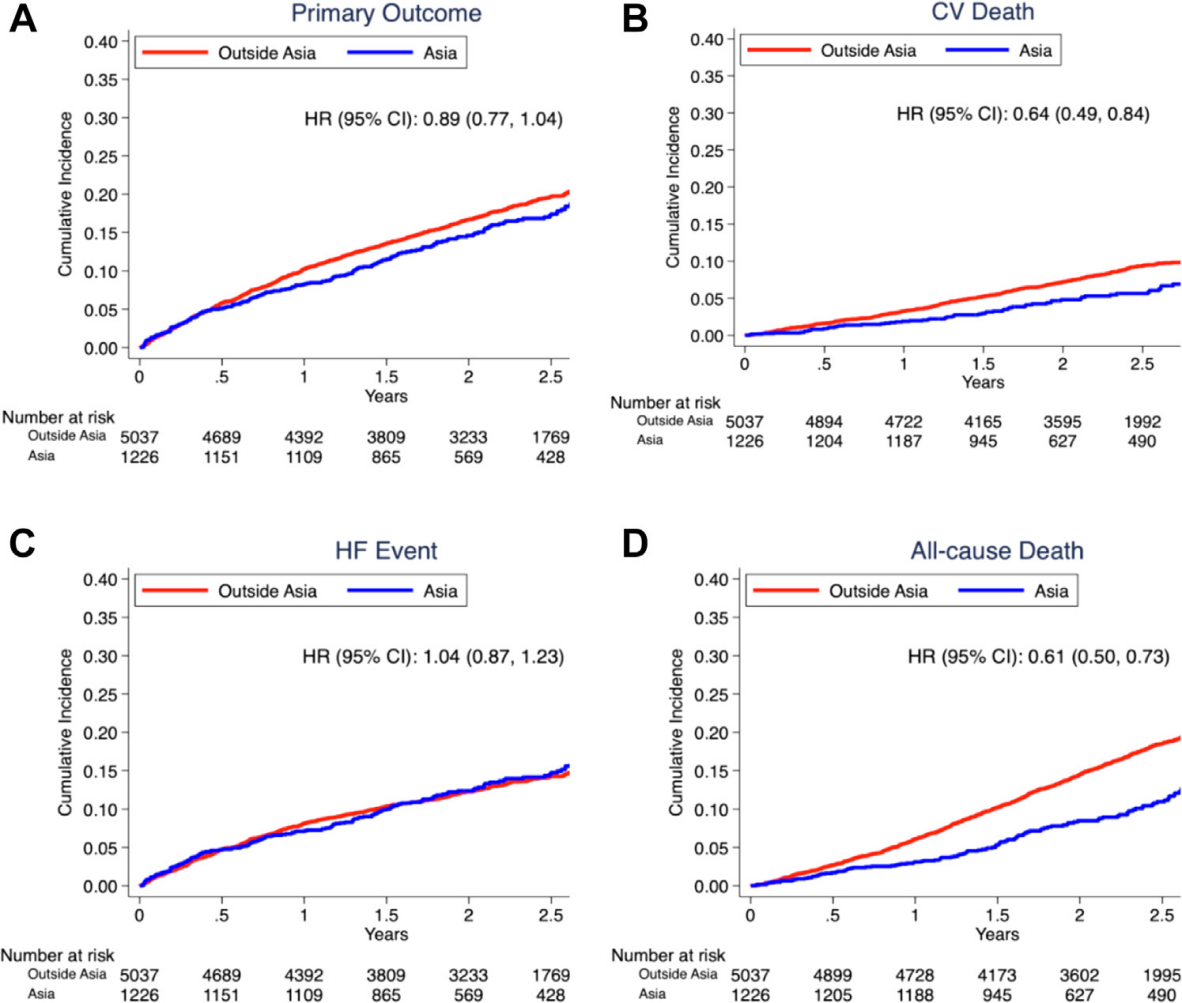
Cumulative Incidence of CV Outcomes According to Geographic Region Cox regression models were used to compare outcomes in those enrolled in Asia vs outside Asia. Unadjusted hazard ratios with participants outside Asia are displayed. The primary outcome was a composite of worsening heart failure (HF) or cardiovascular (CV) death. The cumulative incidence of (A) the primary outcome, (B) cardiovascular death, (C) heart failure events, and (D) all-cause death were estimated with the use of the Kaplan-Meier method in patients enrolled in Asia and outside Asia. Compared with participants outside Asia, those from Asia had lower incidence of CV death and all-cause death, but similar incidence of primary outcome and HF events.

Participants enrolled in Asia and outside Asia had a similar incidence of the primary composite outcome and worsening HF outcomes (including HF hospitalization and urgent HF visits) (Table 2). However, those enrolled in Asia had a lower risk of CV death (HR: 0.64; 95% CI: 0.49-0.84; *P* = 0.001) and all-cause death (HR: 0.61; 95% CI: 0.50-0.73; *P* < 0.001) (Figure 1) compared with those enrolled outside Asia. These differences persisted after adjusting for age, sex, and baseline LVEF, with adjusted HRs (aHRs) of 0.66 (95% CI: 0.51-0.87) and 0.60 (95% CI: 0.49-0.72) for CV death and all-cause death, respectively (Table 2). After adjusting for baseline clinical profiles, those enrolled in Asia still had an almost 50% lower risk of CV death, non-CV death, and all-cause death (Table 2).

**Table 2:** Unadjusted and Adjusted Primary and Secondary Outcomes in Patients in Asia vs Outside Asia

	Events (per 100 py)		Model 1^a^ HR or RR (95% CI) [Ref = Outside Asia]	*P* Value	Model 2^b^ HR or RR (95% CI)^a^ [Ref = Outside Asia]	*P* Value	Model 3^c^ HR or RR (95% CI)^a^ [Ref = Outside Asia]	*P* Value
	Outside Asia (n = 5,037)	Asia (n = 1,226)						
Primary outcome	927 (8.9)	195 (8.0)	0.89 (0.77-1.04)	0.15	0.91 (0.78-1.06)	0.22	0.97 (0.82-1.15)	0.73
HF event	661 (6.3)	162 (6.6)	1.04 (0.87-1.23)	0.69	1.04 (0.88-1.24)	0.64	1.19 (0.98-1.45)	0.08
HF hospitalization	599 (5.7)	148 (6.0)	1.05 (0.88-1.26)	0.59	1.06 (0.88-1.27)	0.55	1.17 (0.95-1.43)	0.14
CV death	428 (3.8)	64 (2.4)	0.64 (0.49-0.84)	0.001	0.66 (0.51-0.87)	0.002	0.56 (0.42-0.75)	<0.001
All-cause death	897 (8.0)	126 (4.8)	0.61 (0.50-0.73)	<0.001	0.60 (0.49-0.72)	<0.001	0.54 (0.44-0.66)	<0.001
Non-CV death	464 (4.1)	61 (2.3)	0.57 (0.43-0.74)	<0.001	0.53 (0.41-0.70)	<0.001	0.52 (0.39-0.69)	<0.001
CV death and recurrent HF events	1,526 (13.6)	346 (13.2)	0.97 (0.81-1.17)	0.75	0.99 (0.82-1.19)	0.90	1.09 (0.90-1.33)	0.39

HF event refers to HF hospitalization and urgent outpatient HF visits.

CV = cardiovascular; HF = heart failure; LVEF = left ventricular ejection fraction; py = person-year; RR = rate ratio.

a Model 1: Unadjusted.

b Model 2: Adjusted for age, sex, baseline LVEF.

c Model 3: Adjusted for age, sex, baseline LVEF, body mass index, NYHA functional class, atrial fibrillation/flutter, stroke, dyslipidemia, type 2 diabetes mellitus, myocardial infarction, hypertension, prior HF hospitalization.

### Outcomes in Asian countries

Within countries of Asia, China and Taiwan had a higher rate of the primary composite outcome compared with Japan (HR: 2.38; 95% CI: 1.58-3.58 and HR: 1.90; 95% CI: 1.31-2.76, respectively). Compared with patients enrolled in Japan, those enrolled in China also had a higher risk of an HF event (HR: 2.41; 95% CI: 1.57-3.72) (Supplemental Figure 1), which was mostly driven by HF hospitalization (HR: 2.53; 95% CI: 1.62-3.95) (Table 3). Similar trends were observed after adjusting for age, sex, and baseline LVEF. The incidence of CV death was similar in China and Japan; however, the incidence of all-cause death was higher in China after adjusting for age, sex, and baseline LVEF (aHR: 2.22; 95% CI: 1.14-4.35). In comparison, those enrolled in Vietnam had a higher risk of CV death (aHR: 3.09 [95% CI: 1.39-6.89]), non-CV death (aHR: 3.35 [95% CI: 1.71-6.59]), and all-cause death (aHR: 3.26 [95% CI: 1.95-5.43]) (Table 3). Patients enrolled in Taiwan had a higher risk of a worsening HF event (aHR: 1.66 [95% CI: 1.10-2.52]), CV death (aHR: 4.39 [95% CI: 2.25-8.56]), and all-cause death (aHR: 2.65 [95% CI: 1.68-4.18]) (Table 3).

**Table 3:** Unadjusted and Adjusted Primary and Secondary Outcomes in Patients in Countries of Asia

	Japan (n = 422)	China (n = 310)	Taiwan (n = 318)	Vietnam (n = 176)
Primary outcome [195 events]				
Events (per 100 py)	57 (5.5)	50 (12.3)	62 (10.0)	26 (6.7)
Unadjusted HR (95% CI)	[Ref]	2.38 (1.58-3.50), *P* < 0.001	1.90 (1.31-2.76), *P* < 0.002	1.25 (0.78-1.99), *P* = 0.36
Adjusted HR (95% CI),^a^	[Ref]	2.42 (1.56-3.76), *P* < 0.001	2.00 (1.38-2.90), *P* < 0.001	1.41 (0.87-2.28), *P* = 0.16
HF event [162 events]				
Events (per 100 py)	49 (4.7)	46 (11.3)	47 (7.6)	20 (5.1)
Unadjusted HR (95% CI)	[Ref]	2.41 (1.57-3.72), *P* < 0.001	1.63 (1.08-2.46), *P* = 0.020	1.10 (0.65-1.86), *P* = 0.72
Adjusted HR (95% CI),^a^	[Ref]	2.30 (1.44-3.66), *P* < 0.001	1.66 (1.10-2.52), *P* = 0.016	1.16 (0.68-1.99), *P* = 0.58
HF Hospitalization [148 events]				
Events (per 100 py)	44 (4.2)	45 (11.0)	39 (6.2)	20 (5.1)
Unadjusted HR (95% CI)	[Ref]	2.53 (1.62-3.95), *P* < 0.001	1.46 (0.94-2.26), *P* = 0.09	1.20 (0.70-2.04), *P* = 0.51
Adjusted HR (95% CI),^a^	[Ref]	2.34 (1.45-3.79), *P* < 0.001	1.46 (0.94-2.28), *P* = 0.09	1.22 (0.71-2.11), *P* = 0.47
All-cause death [126 events]				
Events (per 100 py)	39 (3.5)	15 (3.3)	44 (6.7)	28 (6.8)
Unadjusted HR (95% CI)	[Ref]	1.45 (0.77-2.75), *P* = 0.25	2.36 (1.50-3.71), *P* < 0.001	2.22 (1.35-3.66), *P* = 0.002
Adjusted HR (95% CI),^a^	[Ref]	2.22 (1.14-4.35), *P* = 0.020	2.65 (1.68-4.18), *P* < 0.001	3.26 (1.95-5.43), *P* < 0.001
CV Death [64 events]				
Events (per 100 py)	14 (1.3)	9 (2.0)	29 (4.4)	12 (2.9)
Unadjusted HR (95% CI)	[Ref]	2.21 (0.91-5.42), *P* = 0.08	4.09 (2.10-7.95), *P* < 0.001	2.50 (1.15-5.46), *P* = 0.021
Adjusted HR (95% CI),^a^	[Ref]	2.55 (1.00-6.50), *P* = 0.05	4.39 (2.25-8.56), *P* < 0.001	3.09 (1.39-6.89), *P* = 0.006
Non-CV death [61 events]				
Events (per 100 py)	25 (2.2)	6 (1.3)	15 (2.3)	15 (3.6)
Unadjusted HR (95% CI)	[Ref]	1.02 (0.39-2.63), *P* = 0.97	1.32 (0.68-2.57), *P* = 0.42	1.94 (1.01-3.75), *P* = 0.048
Adjusted HR (95% CI),^a^	[Ref]	2.17 (0.80-5.93), *P* = 0.13	1.52 (0.78-2.97), *P* = 0.22	3.35 (1.71-6.59), *P* < 0.001
Composite of CV death and recurrent HF events [346 events]				
Events (per 100 py)	109 (9.9)	98 (21.9)	98 (14.9)	41 (10.0)
Unadjusted RR (95% CI)	[Ref]	2.68 (1.66-4.32), *P* < 0.001	1.62 (1.02-2.51), *P* = 0.032	1.06 (0.62-1.79), *P* = 0.84
Adjusted HR (95% CI),^a^	[Ref]	2.64 (1.63-4.25), *P* < 0.001	1.69 (1.11-2.59), *P* = 0.015	1.19 (0.70-2.02), *P* = 0.51

HF event refers to HF hospitalization and urgent outpatient HF visits.

CV = cardiovascular; HF = heart failure; LVEF = left ventricular ejection fraction; py = person-year; RR = rate ratio.

a Adjusted for age, sex, baseline LVEF.

### Impact of region on treatment effect of dapagliflozin

**Figure 2:**
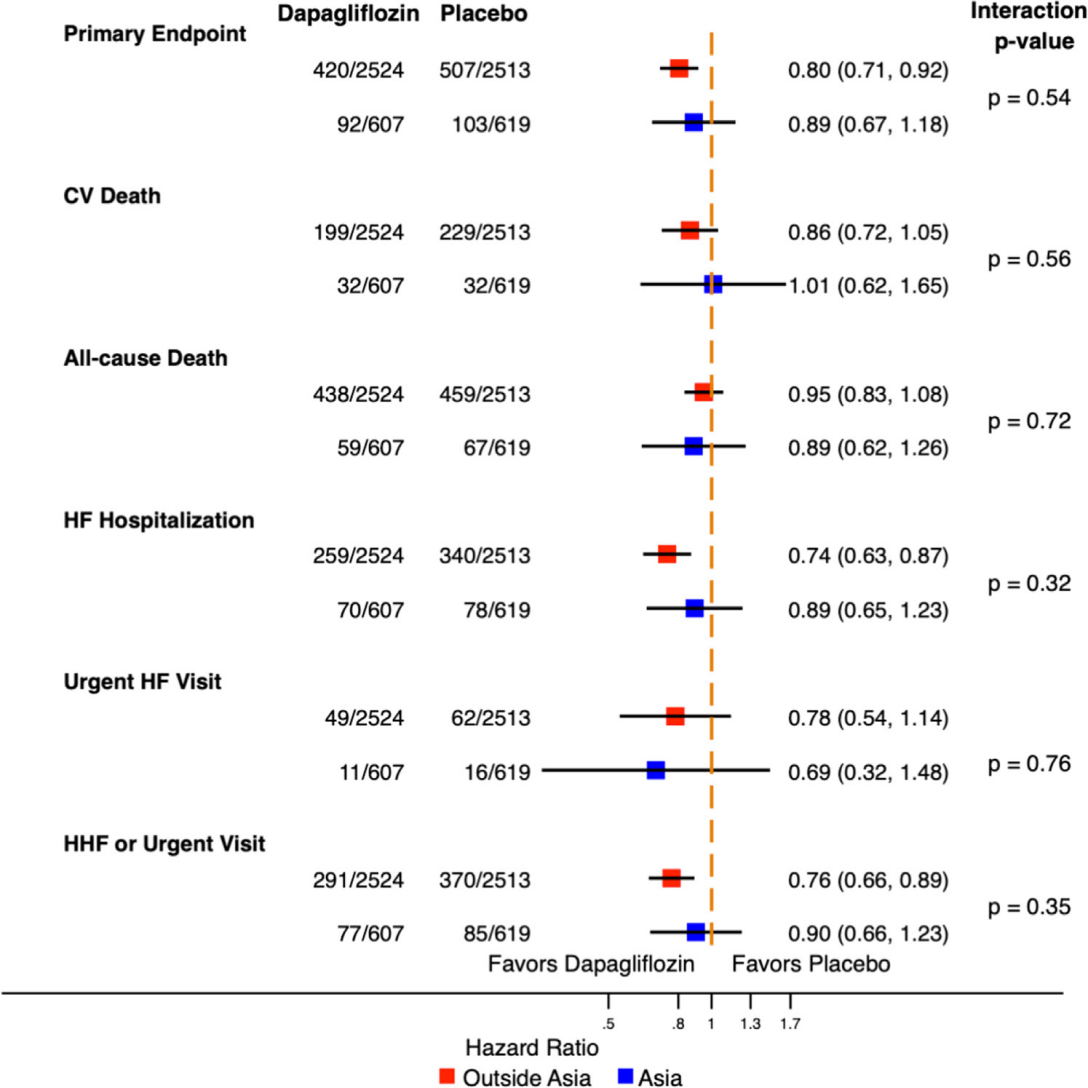
Treatment Effect of Dapagliflozin in Participants According to Geographic Region Cox proportional hazards models without covariates were used to evaluate the effect of dapagliflozin in participants in Asia vs outside Asia. A region-by-treatment interaction term was included in the Cox proportional hazards models to assess the impact of region on the treatment effect of dapagliflozin. Enrollment from Asia did not modify the effect of dapagliflozin on primary or secondary outcomes. The primary outcome was a composite of worsening heart failure (HF) or cardiovascular (CV) death. HHF = hospitalization for heart failure.

Enrollment from Asia did not modify the effect of dapagliflozin on primary outcome (*P*_interaction_ = 0.54), components of primary outcome, or secondary outcomes (*P*_interaction_ >0.32 for all outcomes) (Central Illustration, Figure 2). After adjusting for baseline differences, the treatment effects of dapagliflozin remained similar in both participants from Asia and outside Asia (Supplemental Figure 2).

**Central Illustration:**
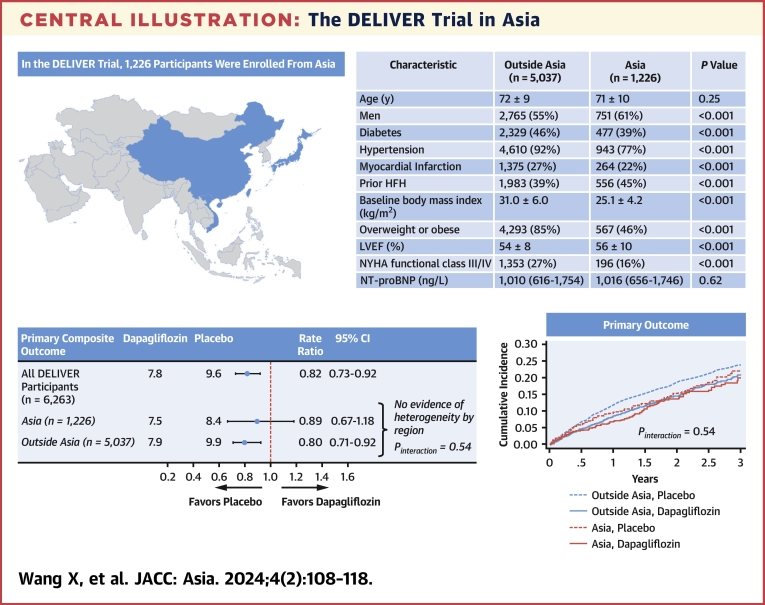
The DELIVER Trial in Asia Select baseline clinical characteristics and primary composite outcome in participants enrolled from Asia vs outside Asia in the DELIVER (Dapagliflozin Evaluation to Improve the Lives of Patients with Preserved Ejection Fraction Heart Failure) trial. Map graphics were created with Microsoft Excel with software powered by Bing. HFH = heart failure hospitalization; LVEF = left ventricular ejection fraction; NT-proBNP = N-terminal pro–B-type natriuretic peptides.

### Regional variations in KCCQ scores

At baseline, participants from Asia had a KCCQ total symptom score (KCCQ-TSS) of 81.2 ± 19.7, compared with a KCCQ-TSS score of 67.5 ± 21.9 in those from outside Asia (*P* < 0.001). Treatment with dapagliflozin resulted in a significant benefit in those enrolled in Asia and outside Asia (Table 4). There was no evidence of region-by-treatment interaction (*P*_interaction_ >0.50 for total symptom score, clinical summary score, and overall summary score) (Table 4).

**Table 4:** Changes in Kansas City Cardiomyopathy Questionnaire Scores From Baseline to 8 Months

	Outside Asia (n = 3430)		Asia (n = 981)		*P*_interaction_
	Change From Baseline to 8 Months	*P* Value	Change From Baseline to 8 Months	*P* Value	
Total symptom score	2.5 (1.4–3.7)	<0.001	2.0 (0.1–3.9)	0.040	0.89
Clinical summary score	2.4 (1.4–3.4)	<0.001	2.1 (0.3–3.8)	0.021	0.82
Overall summary score	2.4 (1.4–3.4)	<0.001	1.3 (-0.4 to 3.0)	0.14	0.55

Values are point changes in Kansas City Cardiomyopathy Questionnaire score (95% CI) unless otherwise indicated.

### Adverse events in Asia vs outside Asia

Data on serious adverse events, adverse events that led to discontinuation of dapagliflozin or placebo, and select other adverse events were collected. Overall, patients enrolled in Asia vs outside Asia had similar rates of adverse events (Supplemental Table 1), with a few exceptions. In the overall patient group (regardless of treatment assignment), those enrolled in Asia had lower rates of adverse events (14.5% outside Asia vs 7.6% in Asia, *P* < 0.001), amputation (0.9% outside Asia vs 0.1% in Asia), and myocardial infarction (2.5% outside Asia vs 1.0% in Asia). In both groups, those randomized to dapagliflozin did not have a higher rate of adverse events compared with those randomized to placebo (Supplemental Table 1). Further, there was no evidence of effect modification by region (*P*_interaction_≥0.07 for all adverse event outcomes) (Supplemental Table 1).

## Discussion

In this subgroup analysis of the DELIVER trial, participants enrolled in Asia had a lower burden of comorbidities, had similar incidence of HF events, but were less likely to experience CV death or all-cause death. Despite these differences, dapagliflozin was well tolerated in both patients in Asia and outside Asia. Further, enrollment from Asia did not modify the effect of dapagliflozin on primary and secondary outcomes.

The differences in baseline characteristics in patients from Asia compared with those from outside Asia were overall consistent with previous studies, with some notable exceptions. In the ASIAN-HF registry, Asian patients were much younger (mean age 68.4 years) compared with those with heart failure with preserved ejection fraction (HFpEF) in Western registries, such as GWTG-HF (Get With The Guideline-Heart Failure, mean age 82 years), SwedeHF (The Swedish Heart Failure Registry, mean age 77.5 years), and OPTIMIZE-HF (Organized Program to Initiate Lifesaving Treatment in Hospitalized Patients With Heart Failure, mean age 75.6 years).^8^ In DELIVER, those enrolled in Asia had a mean age of 71.4 years, compared with a mean age of 71.7 years in those enrolled outside Asia. Consistent with registry data, patients in DELIVER and enrolled in Asia were less likely to have prior myocardial infarction and had lower BMI, with a mean BMI of 25 (Asia) vs 31 (outside Asia). Despite a significantly lower prevalence of overweight/obesity, almost 40% of participants in Asia had diabetes, with the rate as high as 45% in those enrolled from Taiwan. This is consistent with prior findings in ASIAN-HF, where prevalence of diabetes was high despite a lower BMI, suggesting a key role of metabolic derangement in the development of HFpEF^9^ in this patient population.

Although prior studies suggested worse outcomes in certain patients with HF in Asia,^2^^,^^10^ our analyses demonstrated a lower risk of CV death and all-cause death in patients with chronic HF and LVEF >40%. The lower risk of CV death and all-cause death was observed despite a similar risk of HF events. One possible explanation for this observation is the eligibility criteria used in DELIVER, such as the requirement for elevated NT-proBNP, to standardize risk of worsening HF events. In contrast, there were no specific eligibility criteria for other cardiac and noncardiac comorbidities, which may have accounted for the differences in risks of CV death and all-cause death. Regardless, there are notable differences within countries of Asia. Compared with Japan, those from China and Taiwan experienced a higher risk of worsening HF events, CV death, and all-cause death. Those from Vietnam had markedly higher risk of all-cause death, but similar rates of HF events. However, it is worth noting that the overall event rates in patients from Vietnam were low, and thus these cross-country comparisons may be underpowered. Nevertheless, these notable differences reflect the diverse ethnic and sociodemographic backgrounds of people in Asia. For example, patients from Japan are known to have one of the longest life expectancies in the world, and the overall better outcomes are likely a reflection of its socioeconomics and health care infrastructure.^11^ In comparison, even though patients from Vietnam were younger and had lower comorbidity burden compared with other regions in Asia, they still had markedly higher mortality rates. This is consistent with the observations in the ASIAN-HF registry, where Southeast Asians with HFpEF had the highest rates of death or HF hospitalization.^9^ Even after adjusting for baseline comorbidities and demographic factors, Southeast Asians in the ASIAN-HF registry had a 2.7-fold risk of death of HF hospitalizations compared with Northeast Asians, and an almost 4-fold risk of all-cause death.^9^ This higher risk of adverse outcomes warrants further research to understand the underlying social and biological factors driving this risk.

Patients in DELIVER and enrolled from Asia derived similar benefits from dapagliflozin compared with those outside Asia. This is consistent with previously reported analyses in DAPA-HF, which evaluated the effect of dapagliflozin in patients with heart failure with reduced ejection fraction.^12^ In DAPA-HF, 1,096 (23.1%) were enrolled in Asia, with similar event rate of primary composite endpoint compared with those enrolled outside Asia (13.9 per 100 person-years vs 13.4 per 100 person-years). Those enrolled in Asia vs outside Asia also had similar rates of worsening HF events, CV death, all-cause death, and total HF hospitalization and CV death.^12^ In DAPA-HF, dapagliflozin had a consistent effect in reducing the primary endpoint in patients enrolled in Asia vs outside Asia, and was well tolerated in both populations. In EMPEROR-Reduced (Empagliflozin Outcome Trial in Patients with Chronic Heart Failure with a Reduced Ejection Fraction), patients with heart failure with reduced ejection fraction were randomized to receive another sodium glucose co-transporter 2 (SGLT2) inhibitor, empagliflozin, or placebo.^13^ There were 493 (13.2%) patients enrolled in Asia, and the effect of empagliflozin in reducing primary outcome (composite of CV death and HF hospitalization) was more pronounced in those enrolled in Asia (HR: 0.55; 95% CI: 0.38-0.78, compared with HR: 0.69; 95% CI: 0.48-1.01 for those enrolled in North America, HR: 0.73; 95% CI: 0.58-0.94 for those enrolled in Latin America, and HR: 0.94; 95% CI: 0.74-1.18 for those enrolled in Europe).^14^ However, there was no evidence of treatment-by-region interaction for the primary outcome (*P*_interaction_ = 0.10).^14^ Taken together, these data suggest that enrollment from Asia does not modify the efficacy of SGLT2 inhibitors in patients with HF. This further adds to the accumulating evidence that SGLT2 inhibitors are safe and effective in Asian patients.^15^, ^16^, ^17^

Our study highlighted the importance of including Asian patients in clinical trials. Earlier HF trials included few or no Asians.^4^ For example, early HF trials of ACE inhibitors and beta blockers did not enroll any patients from Asia.^4^ In comparison, PARADIGM-HF (Prospective Comparison of ARNI [Angiotensin Receptor–Neprilysin Inhibitor] with ACEi to Determine Impact on Global Mortality and Morbidity in Heart Failure) and PARAGON-HF (The Prospective Comparison of with ARB Global Outcomes in HF with Preserved Ejection Fraction) enrolled 18% and 16% of patients from Asia, respectively.^5^^,^^18^ DELIVER has a high proportion of participants from Asia (20%), making it uniquely suited to evaluate the clinical characteristics and outcomes in a contemporary HFpEF population.^5^

### Study limitations

First, the analyses presented here were post hoc subgroup analyses of a large, randomized trial, and the results should be interpreted as hypothesis generating. Second, although DELIVER enrolled 20% of participants from Asia, the distribution of these participants was not representative of the overall diverse ethnic and sociodemographic background of patients in Asia. For example, DELIVER did not include any patients from India, which has one of the largest populations in the world with a distinct risk profile and outcomes as reported by other studies. Third, as is the case with many subgroup analyses, we have a lower number of participants and events in the Asian subgroup, limiting the statistical power to assess the treatment effect of dapagliflozin in this population. We were restricted to perform the adjusted analysis using a limited model when comparing outcomes between Asian countries due to small number of events. However, our results highlighted the importance of enrolling more patients from Asia, as well as further epidemiological studies to understand the unique characteristics of Asian patients with HF.

## Conclusions

In the DELIVER trial, patients who were enrolled in Asia had lower risks of CV death or all-cause death compared with those enrolled outside Asia. Overall clinical benefits observed in the DELIVER trial were not modified by enrollment from Asia.

Perspectives **COMPETENCY IN MEDICAL KNOWLEDGE:** Asian patients with heart failure with mildly reduced ejection fraction and HFpEF have distinct clinical characteristics compared with non-Asian patients.

**COMPETENCY IN PATIENT CARE:** The clinical benefits of dapagliflozin are not modified by enrollment from Asia. Dapagliflozin should be a foundational therapy for heart failure with mildly reduced ejection fraction and HFpEF in Asian patients.

**TRANSLATIONAL OUTLOOK:** In the DELIVER trial, patients enrolled in Asia had lower risks of CV death and all-cause death, despite a similar risk of HF events. There were also significant intraregional variations among Asian patients. Further studies are needed to evaluate the underlying drivers and contributors to such differences.

## Funding Support and Author Disclosures

AstraZeneca was the sponsor and funder of the DELIVER trial. Dr Wang is supported by a T32 postdoctoral training grant from the National Heart, Lung, and Blood Institute (T32 HL094301), and by the Scott Schoen and Nancy Adams First.In.Women Cardiovascular Fellowship, Mary Horrigan Connors Center for Women’s Health and Gender Biology at Brigham and Women’s Hospital. Dr Lam has received research support from NovoNordisk and Roche Diagnostics; has received consulting fees from Alleviant Medical, Allysta Pharma, Amgen, AnaCardio AB, Applied Therapeutics, AstraZeneca, Bayer, Boehringer Ingelheim, Boston Scientific, Bristol Myers Squibb, CardioRenal, Cytokinetics, Darma Inc, EchoNous Inc, Eli Lilly, Impulse Dynamics, Intellia Therapeutics, Ionis Pharmaceutical, Janssen Research & Development LLC, Medscape/WebMD Global LLC, Merck, Novartis, Novo Nordisk, Prosciento Inc, Quidel Corporation, Radcliffe Group Ltd, Recardio Inc, ReCor Medical, Roche Diagnostics, Sanofi, Siemens Healthcare Diagnostics, and Us2.ai; and is a co-founder and non-executive director of Us2.ai. Dr Vaduganathan has received research grant support, served on advisory boards, or had speaker engagements with American Regent, Amgen, AstraZeneca, Bayer AG, Baxter Healthcare, Boehringer Ingelheim, Chiesi, Cytokinetics, Lexicon Pharmaceuticals, Merck, Novartis, Novo Nordisk, Pharmacosmos, Relypsa, Roche Diagnostics, Sanofi, and Tricog Health, and participates on clinical trial committees for studies sponsored by AstraZeneca, Galmed, Novartis, Bayer AG, Occlutech, and Impulse Dynamics. Dr Kondo receives lecture fees from Abbott Medical Japan LLC, Ono Pharmaceutical Co, Ltd, Otsuka Pharmaceutical Co, Ltd, Novartis Pharma K.K., AstraZeneca K.K., Bristol Myers Squibb Co, and Abiomed Japan K.K. Dr Yang reports travel grants from AstraZeneca. Dr Vinh received financial support as PI of DAPA-HF, DELIVER trials from AZ, and honoraria as speakers from AZ, Boehringer Ingelheim, Servier, Roche, Abbott, and Menarini. Dr Chiang received honoraria from AstraZeneca, Bayer, Boehringer Ingelheim, Daiichi Sankyo, Pfizer, Menarini, MSD, Novartis, Sanofi, and Viatris. Dr Kitakaze reports grants from the Japanese government, Japan Heart Foundation, Japan Cardiovascular Research Foundation, Kowa, Novartis, and Takeda; personal fees from Daiichi Sankyo, Eli Lilly, Otsuka, and Viatris; and grants and personal fees from Ono, Tanabe-Mitsubishi, AstraZeneca, and Boehringer Ingelheim, outside the submitted work. Dr Jhund reported speaker fees from AstraZeneca, Novartis, Alkem Metabolics, ProAdWise Communications, Sun Pharmaceuticals; advisory board fees from AstraZeneca, Boehringer Ingelheim, Novartis; research funding from AstraZeneca, Boehringer Ingelheim, Analog Devices Inc. Dr Jhund’s employer the University of Glasgow has been remunerated for clinical trial work from AstraZeneca, Bayer AG, Novartis, and NovoNordisk; Director Global Clinical Trial Partners (GCTP). Dr Desai reports being the named recipient on institutional research grants to Brigham and Women’s Hospital from Abbott, Alnylam, AstraZeneca, Bayer, and Novartis as well as personal consulting fees from Abbott, Alnylam, AstraZeneca, Avidity Biopharma, Axon Therapeutics, Bayer, Biofourmis, Cytokinetics, GlaxoSmithKline, Medpace, Merck, Novartis, Parexel, River2Renal, Roche, Veristat, Verily, and Zydus. Dr Inzucchi has served on clinical trial committees or as a consultant to AstraZeneca, Boehringer Ingelheim, Novo Nordisk, Merck, Pfizer, and Bayer, and has delivered lectures sponsored by AstraZeneca and Boehringer Ingelheim. Dr De Boer has received research grants and/or fees from AstraZeneca, Abbott, Boehringer Ingelheim, Cardior Pharmaceuticals GmbH, Ionis Pharmaceuticals, Inc, Novo Nordisk, and Roche; and has had speaker engagements with Abbott, AstraZeneca, Bayer, Bristol Myers Squibb, Novartis, and Roche. Dr Martinez receives consulting fees from AstraZeneca. Dr Kosiborod receives consulting fees from Alnylam Pharmaceuticals, Amgen, Applied Therapeutics, AstraZeneca, Bayer, Boehringer Ingelheim, Cytokinetics, Eli Lilly, Esperion Therapeutics, Janssen Global Services, Lexicon Pharmaceuticals, Merck, Novo Nordisk, Pharmacosmos, Sanofi, Vifor Pharma; he also receives grant/contract or travel fees from AstraZeneca and Boehringer Ingelheim. Dr Hernandez has received research grants from American Regent, Amgen, AstraZeneca, Bayer, Boehringer Ingelheim, Merck, Novartis, Somologic and Verily; and has served as a consultant or on the Advisory Board for Amgen, AstraZeneca, Bayer, Boehringer Ingelheim, Boston Scientific, Bristol Myers Squibb, Cytokinetics, Eidos, Intercept, Merck, and Novartis. Dr Claggett reported receiving consulting fees from Alnylam, Cardurion, Corvia, Cytokinetics, Intellia, and Rocket outside of the submitted work. Dr Langkilde is employed by and holds stock in AstraZeneca. Dr McMurray is supported by a British Heart Foundation Centre of Research Excellence Grant RE/18/6/34217. He has received payments through Glasgow University from work on clinical trials, consulting, and other activities from Alnylam, Amgen, AstraZeneca, Bayer, Boehringer Ingelheim, BMS, Cardurion, Cytokinetics, Dal-Cor, GSK, Ionis, KBP Biosciences, Novartis, Pfizer, and Theracos; and personal lecture fees from the Corpus, Abbott, Hikma, Sun Pharmaceuticals, Medscape/Heart.Org, Radcliffe Cardiology, Servier Director, Global Clinical Trial Partners (GCTP). Dr Solomon has received research grants from Alnylam, AstraZeneca, Bellerophon, Bayer, BMS, Cytokinetics, Eidos, Gossamer, GSK, Ionis, Lilly, MyoKardia, National Institutes of Health/National Heart, Lung, and Blood Institute, Novartis, NovoNordisk, Respicardia, Sanofi Pasteur, Theracos, US2.AI and has consulted for Abbott, Action, Akros, Alnylam, Amgen, Arena, AstraZeneca, Bayer, Boehringer Ingelheim, BMS, Cardior, Cardurion, Corvia, Cytokinetics, Daiichi Sankyo, GSK, Lilly, Merck, Myokardia, Novartis, Roche, Theracos, Quantum Genomics, Cardurion, Janssen, Cardiac Dimensions, Tenaya, Sanofi Pasteur, Dinaqor, Tremeau, CellProThera, Moderna, American Regent, Sarepta, Lexicon, Anacardio, Akros, and Valo. All other authors have reported that they have no relationships relevant to the contents of this paper to disclose.
