# Supplementary material for: Effects of Dapagliflozin in Patients in Asia: A Post Hoc Subgroup Analysis From the DELIVER Trial
Source: JACC Asia. 2023 Dec 5;4(2):108–18. doi: 10.1016/j.jacasi.2023.10.005 (PMC10866733; doi:10.1016/j.jacasi.2023.10.005)
Supplement: Supplemental Table 1 and Supplement Figures 1–2 [file mmc1.docx]

**Effects of Dapagliflozin in Patients in Asia: A Post-Hoc Subgroup Analysis From the DELIVER Trial**

Xiaowen Wang, MD, MPH, Carolyn S.P. Lam, MD, Muthiah Vaduganathan, MD, MPH, Toru Kondo MD, PhD, Mingming Yang, MD, PhD, Yaling Han, MD, PhD, Pham Nguyen Vinh, MD, PhD, Chern-En Chiang, MD, PhD, Masafumi Kitakaze, MD, PhD, Zi Michael Miao, MS, Pardeep S. Jhund, MBChB, MSc, PhD, Akshay S. Desai, MD, MPH, Silvio E. Inzucchi, MD, Rudolf A. de Boer, MD, Felipe A. Martinez, MD, Mikhail N. Kosiborod, MD, Adrian F. Hernandez, MD, Brian Claggett, PhD, Anna Maria Langkilde, MD, John J. V. McMurray, MD, Scott D. Solomon, MD

**Supplemental Table 1: Treatment related adverse events in patients enrolled from Asia or outside Asia.**

|  | **Outside Asia**  **N= 5037** | | | **Asia**  **N = 1226** | | | **p-value Asia vs outside Asia** | **Region-by-treatment**  **P-interaction** |
| --- | --- | --- | --- | --- | --- | --- | --- | --- |
|  | Total  N = 5037 | Dapagliflozin  N = 2524 | Placebo  N = 2513 | Total | Dapagliflozin  N = 607 | Placebo  N = 619 |  |  |
| Any serious adverse event | 2250 (44.7%) | 1107 (43.9%) | 1143 (45.6%) | 534 (43.6%) | 254 (41.9%) | 280 (45.2%) | 0.46 | 0.70 |
| Any adverse event that led to drug discontinuation | 283 (5.6 %) | 149 (5.9 %) | 134 (5.3 %) | 80 (6.5 %) | 33 (5.4 %) | 47 (7.6 %) | 0.23 | 0.75 |
| Any adverse event that led to dose interruption | 744 (14.8%) | 361 (14.3%) | 383 (15.3%) | 186 (15.2%) | 75 (12.4%) | 111 (17.9%) | 0.73 | 0.78 |
| Any amputation | 43 (0.9 %) | 19 (0.8 %) | 24 (1.0 %) | 1 (0.1 %) | 0 (0.0 %) | 1 (0.2 %) | 0.004 | -- |
| Any adverse event that potentially placed a patient at risk for lower-limb amputation | 305 (6.1 %) | 147 (5.8 %) | 158 (6.3 %) | 82 (6.7 %) | 41 (6.8 %) | 41 (6.6 %) | 0.41 | 0.07 |
| Any definite or probable diabetic ketoacidosis | 2 (0.0 %) | 2 (0.1 %) | 0 (0.0 %) | 0 (0.0 %) | 0 (0.0 %) | 0 (0.0 %) | 0.49 | -- |
| Any major hypoglycemic event | 10 (0.2 %) | 5 (0.2 %) | 5 (0.2 %) | 3 (0.2 %) | 1 (0.2 %) | 2 (0.3 %) | 0.75 | -- |
| Any serious adverse event or adverse event that led to discontinuation of dapagliflozin or placebo that was suggestive of volume depletion | 63 (1.3 %) | 38 (1.5 %) | 25 (1.0 %) | 11 (0.9 %) | 4 (0.7 %) | 7 (1.1 %) | 0.30 | -- |
| Any renal serious adverse event or adverse event that led to discontinuation of dapagliflozin or placebo | 131 (2.6 %) | 65 (2.6 %) | 66 (2.6 %) | 21 (1.7 %) | 8 (1.3 %) | 13 (2.1 %) | 0.07 | 0.69 |

**Supplemental Figure 1: Cumulative incidence for key outcomes in countries/regions of Asia.**

| 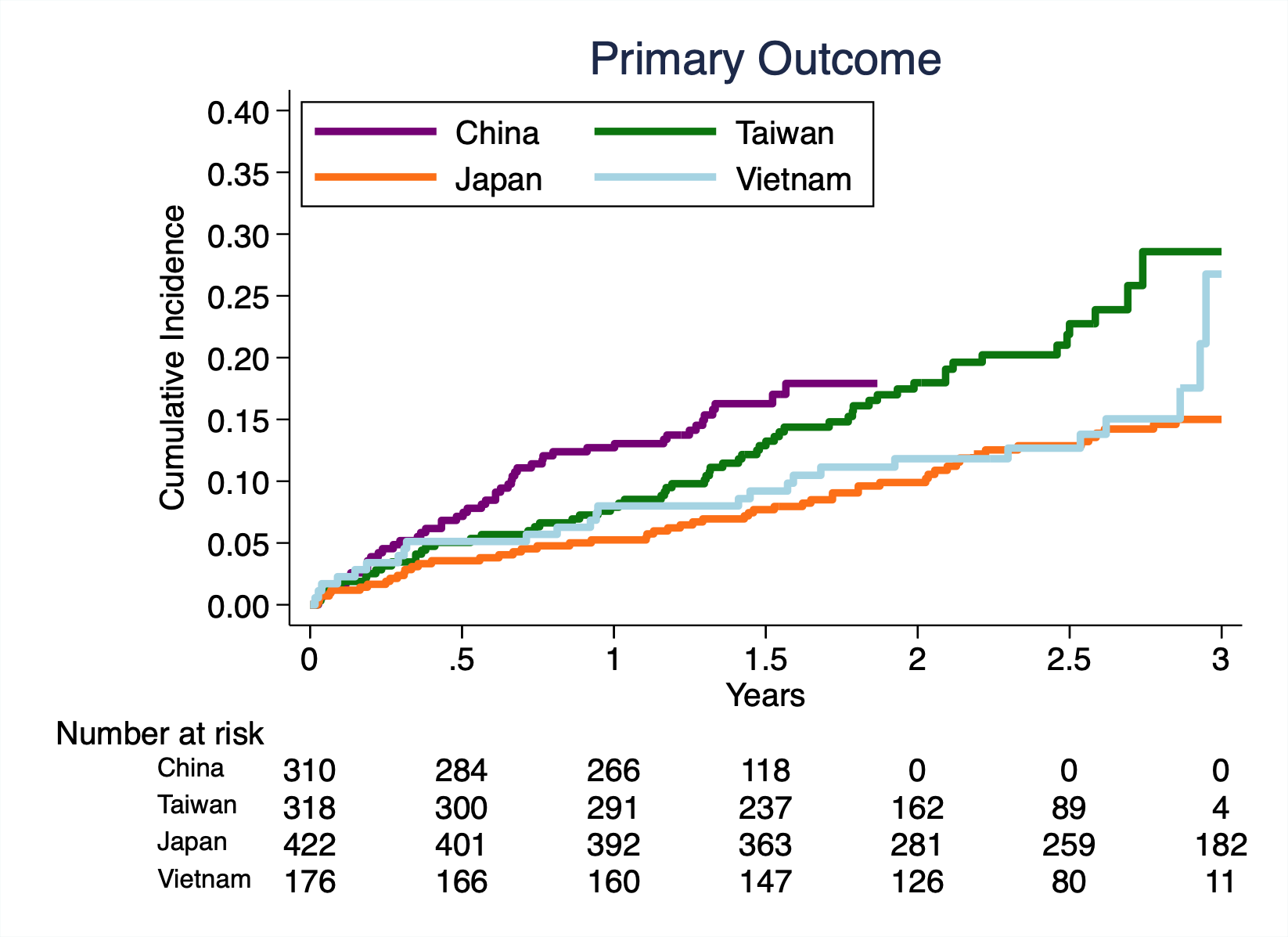 | 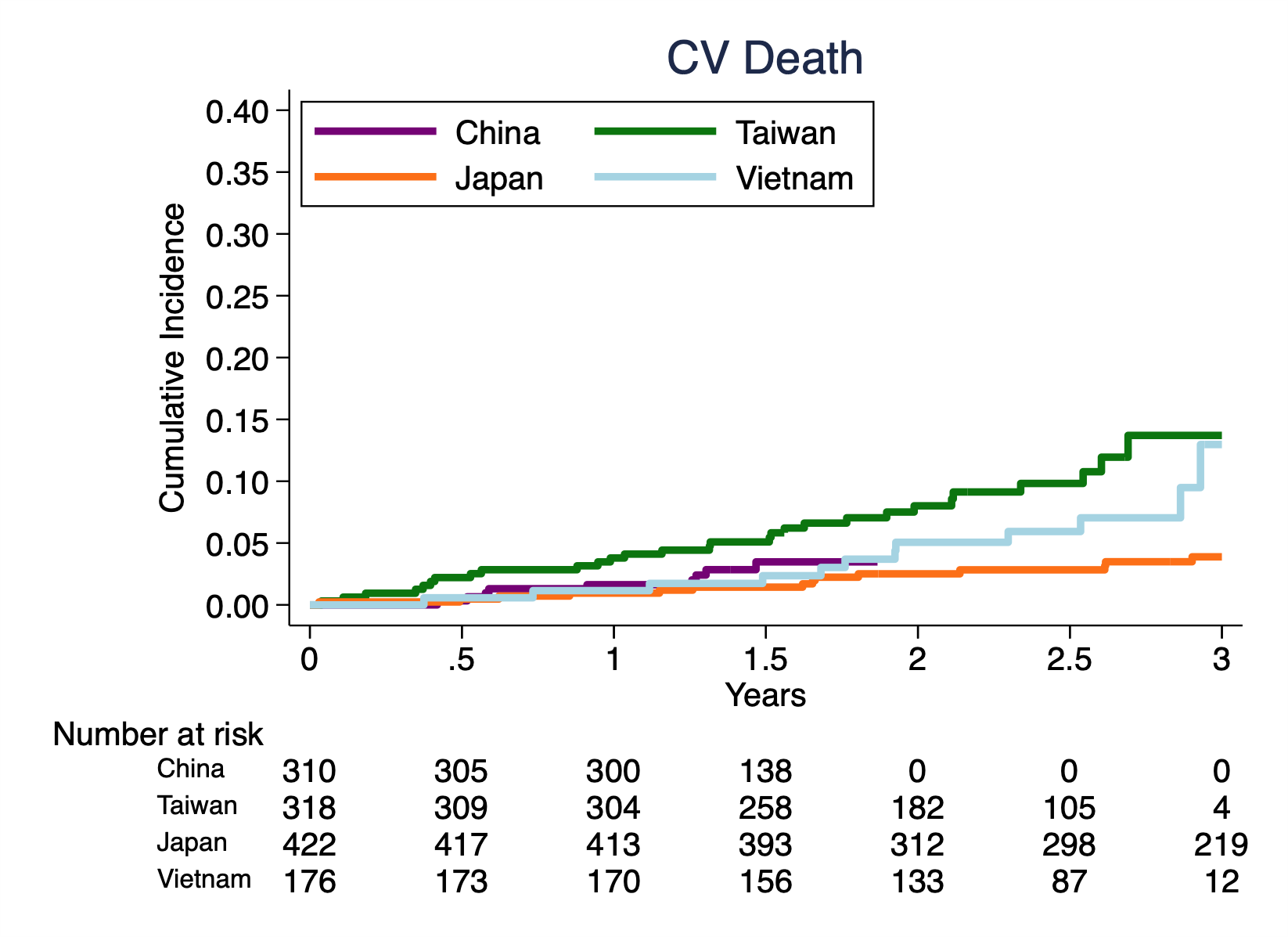 |
| --- | --- |
| 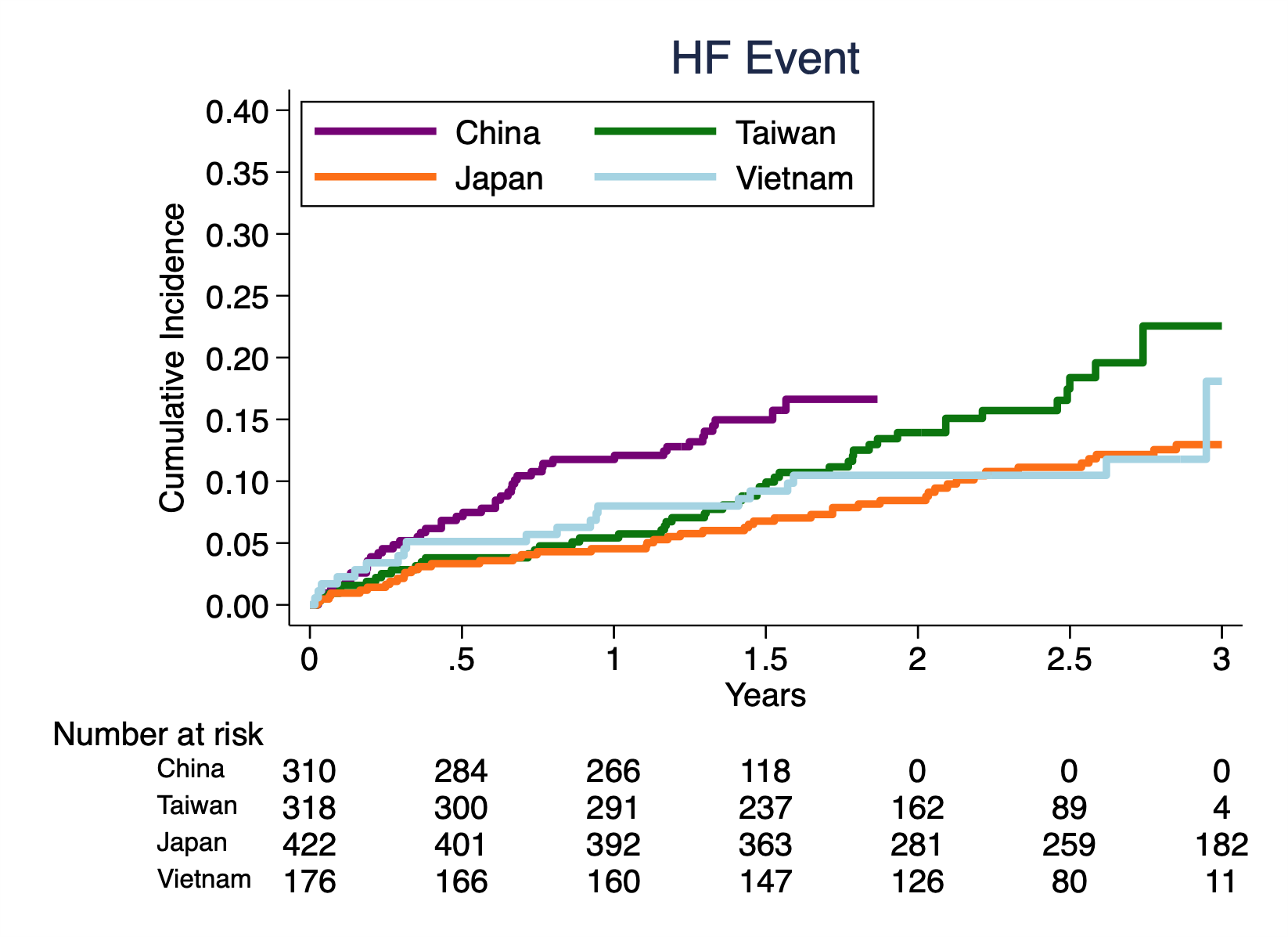 | 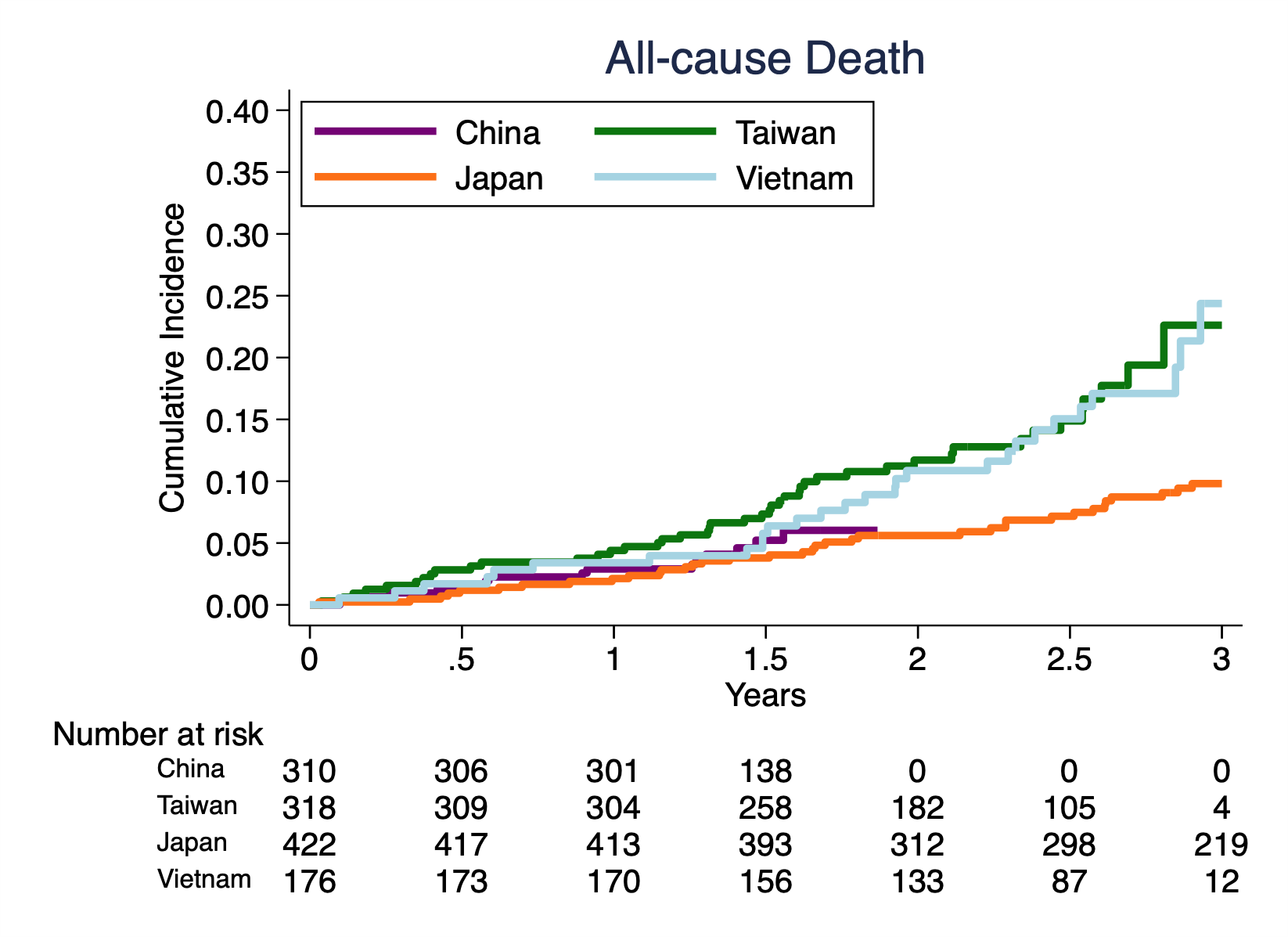 |

The primary outcome was a composite of worsening heart failure (HF) or cardiovascular (CV) death. The cumulative incidence of (A) the primary outcome, (B) CV death, (C) worsening HF events, and (D) all-cause death were estimated with the use of Kaplan-Meier method.

**Supplement Figure 2: Adjusted treatment effect of dapagliflozin in participants according to region.**


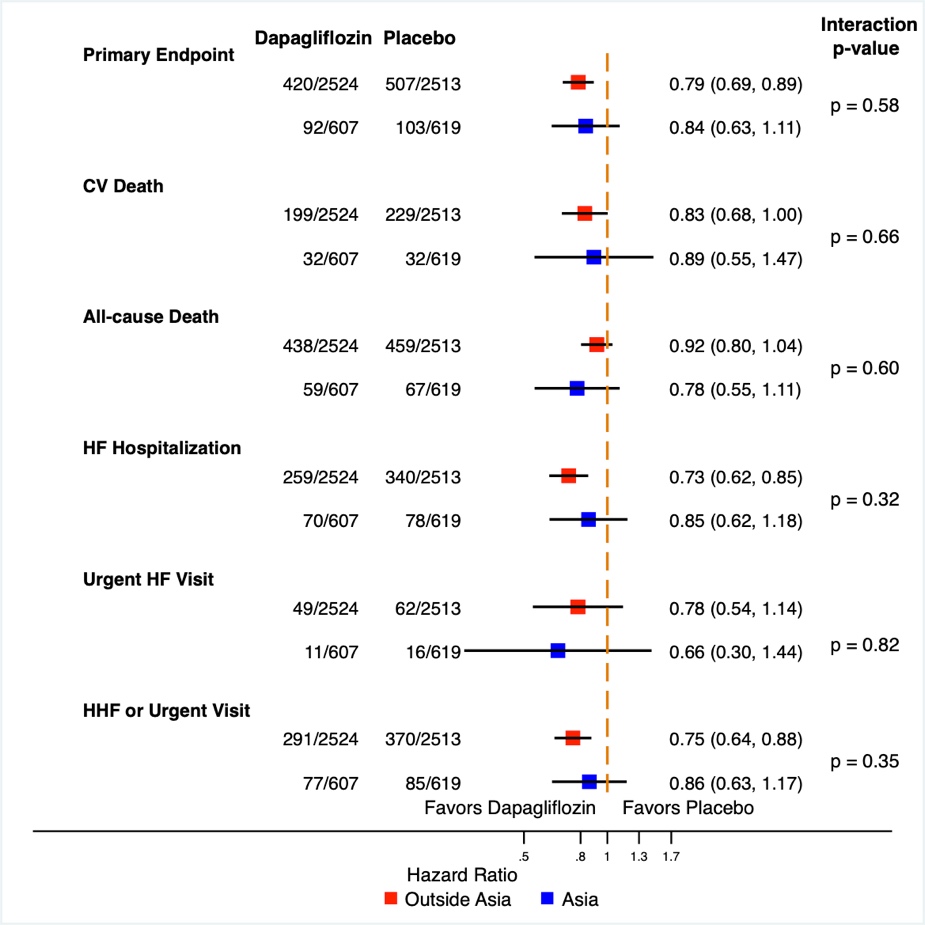


The primary outcome was a composite of worsening heart failure (HF) or cardiovascular (CV) death. Hazard ratios were estimated after adjusting for age, sex, baseline LVEF, body mass index, New York Heart Association class, atrial fibrillation/flutter, stroke, dyslipidemia, type 2 diabetes mellitus, myocardial infarction, hypertension, prior HF hospitalization.

HHF = hospitalization for heart failure.
